# Music does not alter anxiety in patients with suspected lung cancer undergoing bronchoscopy: a randomised controlled trial

**DOI:** 10.3402/ecrj.v3.33472

**Published:** 2016-11-03

**Authors:** Elisabeth Jeppesen, Carsten M. Pedersen, Klaus R. Larsen, Anne Rehl, Karen Bartholdy, Emil S. Walsted, Vibeke Backer

**Affiliations:** 1 Department of Respiratory Medicine, Bispebjerg Hospital, Copenhagen, Denmark; 2 Department of Thoracic Anaesthesiology, Rigshospitalet, Copenhagen, Denmark

**Keywords:** bronchoscopy, music, lung cancer, MusiCure, STAI

## Abstract

**Background:**

The use of music to relieve anxiety has been examined in various studies, but the results are inconclusive.

**Methods:**

From April to October 2015, 160 patients undergoing examination of pulmonary nodules were randomly assigned to MusiCure or no music. MusiCure was administered through earplugs to ensure blinding of the staff and was played from admission to the operating theatre to the end of the bronchoscopy. Spielberger’s State-Trait Anxiety Inventory (STAI) was administered on admission, immediately before bronchoscopy, and on discharge. Secondary outcomes were *p*-cortisol, physiological variables, dosage of sedatives, movements measured by Actigraph, bronchoscopy duration, number of re-examinations, and overall perception of the sounds in the operating theatre measured by Visual analogue scale.

**Results:**

The STAI scores were similar on admission, but after a 10-min wait in the operating theatre, scores varied significantly between patients with and without music, with lower scores in the music group [median (interquartile range, IQR) 35 (18) vs. 43 (25); *p=*0.03]. *Post hoc* multiple regression revealed treatment group as insignificant when adjusting for sex and baseline anxiety. However, there was a significantly more positive perception of the sounds in the operating theatre in the music group (median (IQR) 8.2 (1.8) vs. 5.4 (6.8); *p*<0.0001) and fewer re-examinations in the music group (19.2% vs. 7.7%, *p<*0.032).

**Conclusions:**

Ten minutes with MusiCure does not alter anxiety when adjusting for baseline anxiety and sex. The current study indicates that this field of research has many confounders.

Patients undergoing bronchoscopy may be anxious because of the invasive procedure and exhibit fear of pain, breathing difficulties, loss of control, and fear of the unknown, for instance (1). Anxiety raises the level of hormones, such as cortisol, and elevates blood pressure, heart rate, and respiration (2–5).

Sedation with midazolam and fentanyl effectively relieves patients’ anxiety during bronchoscopy (6–8). However, with a higher dose of sedatives comes an increased risk of respiratory depression (9, 10). Accordingly, it is relevant to investigate the other means of relieving anxiety in patients with reduced lung function (9, 11–13).

Previous studies report contradictory findings regarding the effects of music on patients undergoing bronchoscopy. In one randomised trial, the music group (new wave music) reported significantly greater comfort and experienced less coughing; there were no significant differences in physiological parameters, dose of sedative, or dyspnoea (14). In contrast, another randomised trial found no significant difference between the music group (piano improvisations) and the control group on state and trait anxiety, using Spielberger’s State-Trait Anxiety Inventory (STAI), or on supplemental administration of midazolam (15). Finally, one randomised trial detected a significant difference between the music group (ambient relaxation music) and the control group in blood pressure and heart rate following bronchoscopy. However, no significant difference was observed in the duration of the bronchoscopy or in the patients’ overall feelings about the procedure (16).

In a recent meta-analysis, which included these three studies published in English and an additional four studies published in Chinese, it was not possible to reach any conclusions regarding the effects of music on anxiety during bronchoscopy due to different instruments being used for measurement and lack of reporting of the standard deviation. Nevertheless, the authors concluded that music is an effective way of reducing patients’ blood pressure and heart rate during bronchoscopy (17).

The effects of music in relation to endoscopic procedures were the topic of a systematic review that includes the aforementioned randomised studies. The authors concluded that music decreases anxiety and heart rate overall, but in a subanalysis found that it had no significant effect regarding bronchoscopy (18). Moreover, a review including just one of the bronchoscopy studies investigating the effect of music on state anxiety scores concluded that music reduces anxiety in normal care delivery but not in unpleasant procedures, such as bronchoscopy (19). Two other systematic reviews investigating the effect of music on preoperative anxiety concluded that music has an effect on pre-procedural state anxiety but has no significant effect on physiological variables (20, 21). Finally, a systematic review studying perioperative settings not including bronchoscopy concluded that music had a positive effect on patients’ pain and anxiety in approximately half the reviewed studies (22).

MusiCure is music developed by the Danish composer Niels Eje. To date, the use of MusiCure has been studied mostly post-operatively and within the field of cardiac surgery. The endpoints of these studies vary, but most of the articles favour MusiCure. In two studies on adult patients with anxiety as endpoint, one shows a significant effect on anxiety and the other shows no effect (23–30).

Due to the inconsistencies in the reported findings, the aim of this study was to measure the effect of MusiCure on bronchoscopy-related anxiety. We hypothesised that MusiCure reduces bronchoscopy-related anxiety.

## Methods

### Design

**Fig. 1:**
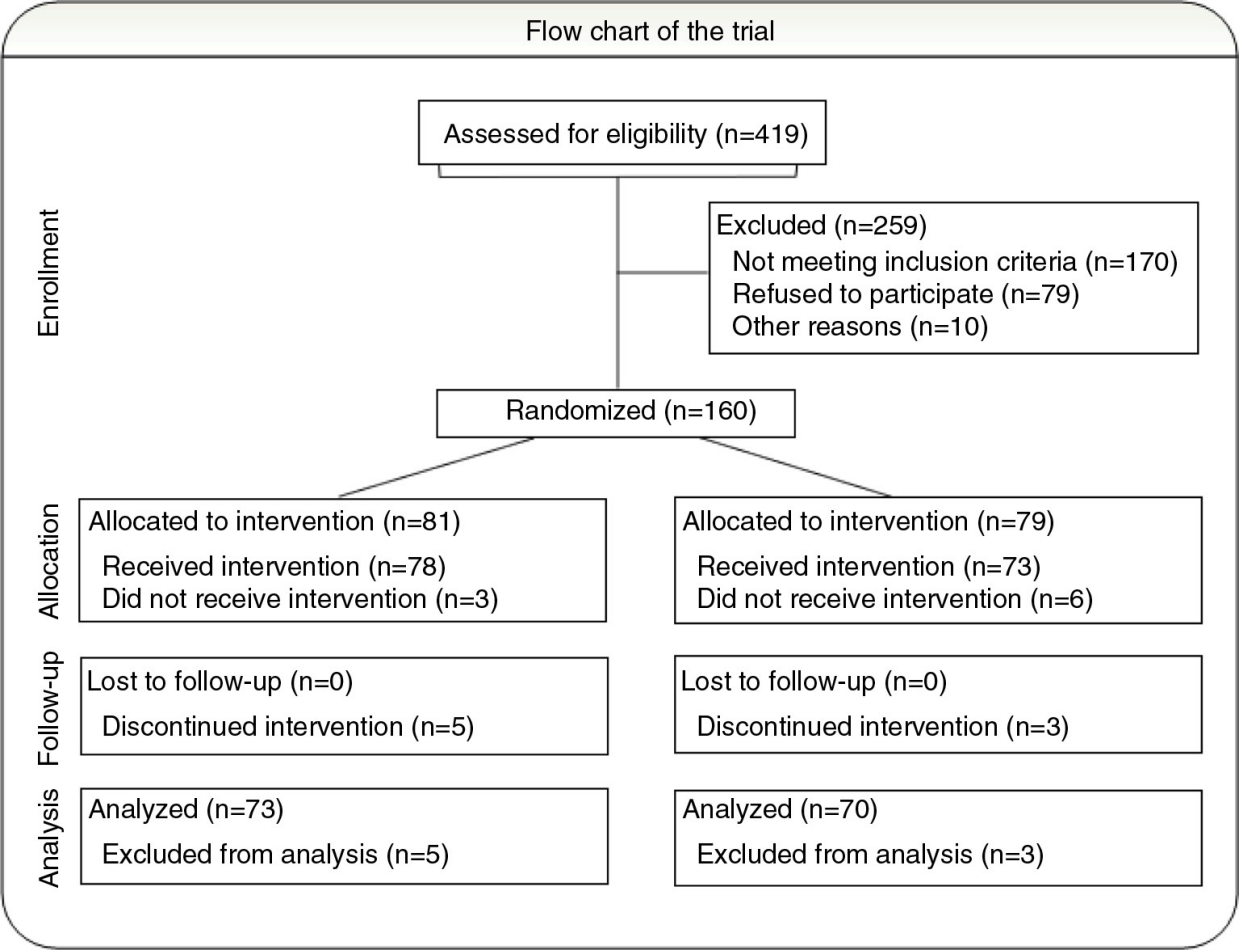
In the study period 160 patients with pulmonary nodules were randomly assigned to either MusiCure or as control group. Nine patients dropped out after randomisation, leaving 78 participants to receive MusiCure and 73 patients in the control group.

This study is a randomised, investigator-blinded, controlled trial. A total of 419 patients were scheduled for bronchoscopy for pulmonary nodules in the study period. Of the 239 patients who met the eligibility criteria, 79 declined participation (Fig. 1). Patients were eligible for participation if they had no hearing or memory impairment and were able to understand, read, and write Danish, and complete the questionnaire unaided. In addition, modes of sedation were restricted to only midazolam and/or fentanyl, and no invasive diagnostics except bronchoscopy should be performed. The study was approved by the Danish Ethics Committee (H-3-2014-065).

### Study procedures

Patients subject to examination of pulmonary nodules were informed about the study when they were scheduled for bronchoscopy at the outpatient clinic at Bispebjerg University Hospital, Copenhagen. Two authors were responsible for patient enrolment, and written informed consent was obtained from 160 patients on admission. Subsequently, blood pressure, heart rate, respiratory rate, and oxygen saturation were recorded, and a blood sample was taken from the patient’s peripheral venous catheter for analysis of plasma cortisol. Participants also completed the STAI. On admission to the operating theatre, blood pressure, heart rate, respiratory rate, and oxygen saturation measurements were repeated. To ensure blinding to the staff, those performing the bronchoscopy left the room, and a staff member not participating in the bronchoscopy opened the sealed envelope with the randomised treatment: MusiCure or control (no sound). Patients were told not to disclose the allocation. This staff member also fitted the earplugs into the patient’s ears and adjusted the volume on the mp3 player (SanDisk, Sansa Clip+) for the patients randomised to MusiCure and applied sensors recording movement (Actigraph, GT3X+) to the patient’s wrists, ankles, head, and hip. The staff performing the bronchoscopy then re-entered the operating theatre and prepared the bronchoscopy with the patient lying in the supine position while being exposed, through earplugs, to either MusiCure (treatment) or no sound (control). After 10 min, blood pressure, heart rate, respiratory rate, and oxygen saturation were recorded, and the participants completed the State part of the STAI. Afterwards, 5 mg of midazolam was administered and supplemented with 50 µg of fentanyl if endobronchial ultrasound (EBUS) was expected. Patients were supplied with midazolam and fentanyl until they appeared comfortable. Throughout the bronchoscopy, blood pressure, heart rate, respiratory rate, and oxygen saturation were monitored every 15 min. A blood sample was taken from the patient’s peripheral venous catheter for analysis of *p*-cortisol 15 and 60 min after termination of the bronchoscopy. Plasma cortisol was analysed with competitive electrochemiluminescence immunoassay (ECLIA) (Cobas 8000, e602 module, Roche). On discharge, approximately 60 min after bronchoscopy, patients completed the STAI and a visual analogue scale (VAS) where they indicated their overall perception of the sounds in the operating theatre. After 14 days, any patient re-examination was registered.

### Outcome measures

Primary outcome was STAI (form Y), which consists of 40 self-reported items including 20 assessing state-anxiety and 20 assessing trait-anxiety. The scores of each item vary from 1 to 4, and each inventory has a minimum score of 20 and a maximum score of 80. Higher scores indicate higher levels of anxiety (31–33).

The sample size was set at 160, estimating a 20% drop-out and using a significance level of 0.05 and a power of 80%. The standard deviation was set at 10.4 for STAI-State, and a count of 5 was interpreted as a clinically relevant difference (15, 31, 33).

### Statistics

Normally distributed continuous variables were compared using the *t*-test. When not normally distributed, the Mann–Whitney *U*-test was used. The χ^2^ test was used for categorical variables. STAI and VAS scores were analysed using the Mann–Whitney *U*-test, and STAI scores were also analysed using multiple regression. The number of re-examinations was analysed with the χ^2^ test and repeated measurements, such as cortisol, with the paired samples *t*-test. Statistical analyses were completed using Statistical Package for the Social Sciences (SPSS) version 23.0 (Chicago, IL). Participants were randomised by SPSS in blocks of 10 to MusiCure or no music.

## Results

From April to October 2015, 160 participants were randomly assigned to one of the two treatment arms; 9 patients (5.6%) dropped out after randomisation, leaving 78 participants to receive MusiCure and 73 participants in the control group. Reasons for dropping out included having the planned bronchoscopy changed ‘in theatre’ by the physician (*n*=4); 3 of these individuals underwent percutaneous biopsy and 1 underwent endoscopic ultrasound (EUS) in addition to bronchoscopy. Three participants withdrew consent, one participant was withdrawn by the physician due to time pressure, and one participant received music by mistake, although she had been allocated to the no-music group. No clinically significant differences were observed between those who were treated and those who dropped out.

Throughout the course of the study, bronchoscopies were performed by nine different physicians assisted by 12 different nurses. No statistically significant differences were found between the music and the control group regarding attending staff and procedures performed during bronchoscopy.

The STAI scores at the time of admission were similar in the two groups, whereas a significant difference in the STAI-State score [median (interquartile range, IQR) 35 (18) vs. 43 (25); *p*=0.03, *r*=−0.18] was observed between the music and the control group before bronchoscopy, with lower scores in the music group. These differences were no longer seen before discharge, although the scores in both groups were found to be at a lower level [median (IQR) 40 (19) vs. 31 (17), *p<*0.0001 and 37 (19) vs. 30 (13); *p<*0.0001] (Table 1). However, at the time of discharge, the STAI-Trait differed significantly between the two groups (Table 1), whereas the change from 32 to 34=2 versus 0 was not significant (Table 2).

**Table 1:** Comparison of STAI scores

	On admission			After 10 min with or without music, immediately bronchoscopy			On discharge		
						
	Controls	Music	*p*	Controls	Music	*p*	Controls	Music	*p*
STAI-State	40 (19), *N=*70	37 (19), *N=*76	0.3	43 (25), *N=*70	35 (18), *N=*73	0.03*	31 (17), *N=*69	30 (13), *N=*71	0.5
STAI-Trait	32 (15), *N=*70	30 (10), *N=*75	0.3	–	–		34 (14), *N=*66	30 (12), *N=*68	0.02*

Data are expressed as median (interquartile range, IQR); STAI, Spielberger’s State-Trait Anxiety Inventory.

**Table 2:** Spielberger’s State-Trait Anxiety Inventory (STAI) post- and pre-differences

	Change		
		
Interval of STAI measurements	Controls	Music	*p*
STAI-Trait on admission STAI-Trait on discharge	0.5 (4.75)	0.0 (4.0)	0.5

STAI-State on admission STAI-State immediately before bronchoscopy: after 10 min with/without music	0.5 (5.5)	−1.0 (7.0)	0.05*

STAI-State on admission STAI-State on discharge	−6 (12.0)	−6 (12.0)	0.5

STAI-State immediately before bronchoscopy: after 10 min with/without music STAI-State on discharge	−6 (12.0)	−3 (9.25)	0.2

Data are expressed as median (interquartile range, IQR).

On further exploring the apparent effect of MusiCure, a multiple regression analysis revealed that treatment group was not a significant predictor of STAI-State level in the operating theatre when adjusting for sex, STAI-Trait, and baseline STAI-State which, in contrast, were all significant predictors of STAI-State level in the operating theatre (Table 3).

**Table 3:** Linear model of predictors of STAI-State immediately before bronchoscopy, with 95% bias corrected and accelerated confidence intervals reported in parentheses

	*b*	SE B	β	*p*
Model 1				
Constant	41.26 (37.95 to 44.56)	1.67		<0.0001*
Group	−5.31 (−9.94 to −0.68)	2.34	−0.19	0.025*
Model 2				
Constant	0.81 (−3.45 to 5.08)	2.16		0.706
Group	−1.17 (−3.09 to 0.76)	0.98	−0.04	0.234
STAI-State at arrival	0.87 (0.77 to 0.96)	0.05	0.80	<0.0001*
STAI-Trait at arrival	0.17 (0.07 to 0.28)	0.05	0.13	0.001*
Gender	−2.38 (−4.41 to −0.35)	1.03	−0.09	0.022*

Model 1: *N=*143, *R*^2^=0.04; Model 2: *N*=136, *R*^2^=0.84.

**Fig. 2:**
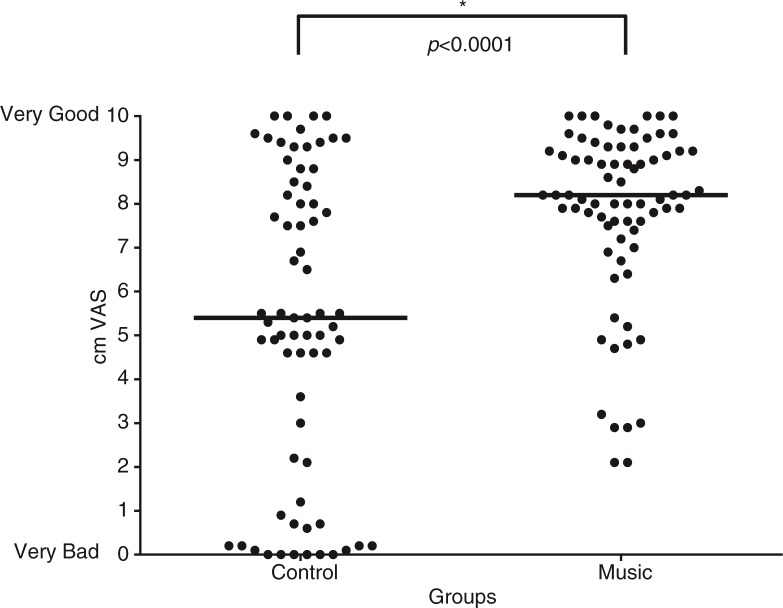
Patients’ overall perception of the sounds in connection with the procedure. The horizontal lines represent the median within the group.

The median VAS score at discharge illustrating the patient’s overall perception of the sounds in the operating theatre showed a highly significant difference between the music and no-music groups [median (IQR) 8.2 (1.8) vs. 5.4 (6.8); *p*<0.0001, *r=*0.36] (Fig. 2).

Significantly more patients required re-examination in the control group compared with the music group (19.2 and 7.7%, respectively; *p<*0.032), with a 2.49 times higher odds ratio of re-examination among those in the no-music group.

No significant differences were found in bronchoscopy duration, sedatives, or amount of movement. Neither were there differences in physiological variables or in plasma cortisol levels.

## Discussion

Music during operative procedures and investigative examinations is not harmful and is inexpensive to administer. It is, therefore, interesting to find out whether it can reduce bronchoscopy-related anxiety. In this study, a lower degree of anxiety after listening to MusiCure for 10 min before bronchoscopy was found in the MusiCure group compared with the control group. However, taking sex and baseline anxiety into consideration, MusiCure had no effect on the level of anxiety. In the current analysis, the factors of importance were baseline STAI-State, STAI-Trait, and sex because they were significant predictors of STAI-State in the operating theatre (Table 3).

A previous study found that music did not reduce anxiety in patients undergoing bronchoscopy (15), and that pre- and post-bronchoscopy scores did not differ significantly. In the current study, however, scores on discharge were significantly lower. The lower values might be because patients in the current study received the bronchoscopy results several days later, whereas those in the previous study completed STAI while waiting for the results of the bronchoscopy. In addition, the lower STAI-State score at discharge might also reflect the fact that each patient received midazolam, whereas in the above-mentioned study, its use was restricted to few patients.

STAI-Trait should be stable over time (33). Colt et al. found a significant decrease in both groups but a non-significant difference between the groups (15). We found a non-significant change but a significant difference between the groups at discharge. Colt et al. explained the change as a test-taking artefact known as the ‘Windle Effect’ (15). This effect was not present in our study, but we were surprised at the significant difference in Trait on discharge. This could be due to an undetected significant difference in Trait on admission.

There are limitations in this study regarding the dose of music. Are 10 min enough to relieve anxiety? Another limitation to be considered is that researcher-selected music does not take into account the patients preference (20, 21).

No differences were found during the current study in physiological variables. This is in line with two systematic reviews assessing the effect of music on preoperative anxiety (20, 21). Previous studies using music during bronchoscopy have found significant differences in blood pressure and heart rate (16, 17). Sample size, differences in cultural upbringing, severity of the disease, type of music, sex, and level of anxiety could influence the effect. However, this is unclear. A study assessing haemodynamic reactions to acute psychological stress in smokers versus ex-smokers and non-smokers found that smokers had significantly smaller systolic and diastolic blood pressure reactions to acute stress than did non-smokers and ex-smokers. Smokers and ex-smokers had lower heart rate reactivity to stress compared with non-smokers (34). This could be a confounder in our study because smoking is a major cause of lung cancer, and our study group patients were undergoing bronchoscopy to determine whether their nodules indicated lung cancer.

In the current study, a lower re-examination rate was found in the music group. This is difficult to explain because there were no differences in the bronchoscopy duration or movements measured by Actigraph. One explanation for a higher frequency of re-examination could be more frequent coughing during bronchoscopy. There were no sensors on the thorax. A previous study reported less coughing when patients listened to music while undergoing bronchoscopy (14). Confounders to movement could be active holding of the patient’s head and hands by the staff, facilitating the bronchoscopy. There was an uneven count of earlier bronchoscopies between the groups (Table 4). Although this count also included bronchoscopies dating back several years, it could be the reason for the differences in re-examination rate.

**Table 4:** Baseline characteristics

	All	Controls	Music	*p*
*N* (female/male)	151 (73/78)	73 (37/36)	78 (36/42)	0.6
Age in years^a^	63.7 (11.6)	64.5 (11.6)	63.0 (11.7)	0.5
Height in cm^a^	172.4 (9.3)	172.5 (9.7)	172.3 (8.9)	0.9
Weight in kg^a^	76.0 (18.9)	76.6 (20.9)	75.5 (16.8)	0.7
BMI^a^	25.4 (5.4)	25.5 (5.7)	25.4 (5.1)	0.9
FEV_1_ percent of predicted^a^	75.0 (22.3)	74.0 (23.0)	76.0 (21.8)	0.6
FEV_1_/FVC ratio^a^	68.4 (12.1)	67.6 (12.6)	69.2 (11.6)	0.4
First examination/re-examination	135/16	68/5	67/11	0.1
Systolic blood pressure^a^	139 (23)	142 (25)	135 (20)	0.1
Diastolic blood pressure^a^	82 (13)	82 (14)	81 (13)	0.5
Mean arterial pressure^b^	100 (18)	98 (17)	100 (18)	0.6
Heart rate^a^	77 (14)	78 (12)	75 (15)	0.2
Plasma cortisol^b^ (nmol/L)	343 (152)	356 (139)	337 (168, 5)	0.3
Baseline anxiety				
State^b^	39 (20)	40 (19)	37 (19)	0.3
Trait^b^	31 (13)	32 (15)	30 (10)	0.3

a Data are expressed as mean (SD)

b data are expressed as median (interquartile range, IQR).

Patients who received MusiCure experienced the sounds in the operating theatre as significantly more pleasant than did the patients in the no-music group. This could be consistent with the findings of greater subjective comfort in the study by Dubois et al. (14). The control group lacked stimulus, and the positive experience of the sound environment in the music group could also be due to distraction (35). That the control group may have reflected less on the sound, could explain the great diversity in this group.

*p*-Cortisol decreased significantly from admission to 15 min after termination of bronchoscopy and also from 15 to 60 min after. There were no significant differences in *p*-cortisol between treatment groups or sexes on admission, at 15 or 60 min after bronchoscopy. It is interesting that there were no statistically significant differences between the sexes because women reported significantly higher anxiety on STAI-State at all three times in this study. *p*-Cortisol values after termination of bronchoscopy could be confounded because all patients received midazolam and most were also sedated with fentanyl, both of which appear to have an impact on *p*-cortisol levels (36–38).

## Conclusion

In this investigator-blinded, randomised, controlled trial examining the effect of music on anxiety in patients undergoing bronchoscopy, 10 min with MusiCure did not alter anxiety when adjusting for baseline anxiety and sex. The current study indicates that this field of research has many confounders.

Implications for future studies could be to expand the intervention to minimum 20 min and also to include a second treatment group with self-selected music.
